# Evaluation of the Factors Affecting the Disintegration under a Composting Process of Poly(lactic acid)/Poly(3-hydroxybutyrate) (PLA/PHB) Blends

**DOI:** 10.3390/polym13183171

**Published:** 2021-09-18

**Authors:** Magdalena L. Iglesias-Montes, Michelina Soccio, Francesca Luzi, Debora Puglia, Massimo Gazzano, Nadia Lotti, Liliana B. Manfredi, Viviana P. Cyras

**Affiliations:** 1 Instituto de Investigaciones en Ciencia y Tecnología de Materiales (INTEMA), Facultad de Ingeniería, Universidad Nacional de Mar del Plata—Consejo de Investigaciones Científicas y Técnicas (CONICET), Mar del Plata 7600, Argentina; ml.iglesiasmontes@gmail.com (M.L.I.-M.); lbmanfre@fi.mdp.edu.ar (L.B.M.); 2 Department of Civil, Chemical, Environmental and Materials Engineering, University of Bologna, 40131 Bologna, Italy; m.soccio@unibo.it (M.S.); nadia.lotti@unibo.it (N.L.); 3 Civil and Environmental Engineering Department, UdR INSTM, University of Perugia, 05100 Terni, Italy; francesca.luzi@unipg.it (F.L.); debora.puglia@unipg.it (D.P.); 4 Institute of Organic Synthesis and Photoreactivity, National Research Council, 40129 Bologna, Italy; massimo.gazzano@isof.cnr.it

**Keywords:** poly(lactic acid), poly(hydroxybutyrate), plasticizer, biobased, biodegradation, compostability

## Abstract

The overall migration behavior and the disintegration under composting conditions of films based on plasticized poly(lactic acid)/poly(3-hydroxybutyrate) (PLA-PHB) blends were studied, with the main aim of determining the feasibility of their application as biodegradable food packaging materials. The role of composition in the disintegration process was evaluated by monitoring the changes in physical and thermal properties that originated during the degradation process. PLA and PHB were blended in two weight ratios with 15 wt% of tributyrin, using a Haake mixer and then compression molded into ~150 μm films. We found that the migration level of all of the studied blends was below check intended meaning retained in non-polar simulants, while only plasticized blends could withstand the contact with polar solvents. The disintegration of all of the materials in compost at 58 °C was completed within 42 days; the plasticized PHB underwent the fastest degradation, taking only 14 days. The presence of the TB plasticizer speeded up the degradation process. Different degradation mechanisms were identified for PLA and PHB. To evaluate the annealing effect separately from bacteria degradation, the influence of temperature on materials in the absence of a compost environment was also studied. With the increasing time of degradation in compost, both melting temperature and maximum degradation temperature progressively decreased, while the crystallinity degree increased, indicating that the samples were definitely degrading and that the amorphous regions were preferentially eroded by bacteria.

## 1. Introduction

The incentive around the packaging industry to create both recyclable and compostable packaging, and the need to find solutions that provide good performance at a reasonable cost, are increasing day by day. To resolve this, much attention has been paid to the development of biodegradable polymeric materials. In this way, blends of poly(lactic acid) (PLA) and poly(3-hydroxybutyrate) (PHB) have been extensively studied over the last two decades for packaging applications [1,2,3,4,5,6,7]. This is due to the synergic effect that occurs when both polymers are mixed, that is, an improvement in their properties. The poor processability and high brittleness of PHB are perhaps the principal drawbacks that limit its industrial application; however, blending it with PLA represents an interesting solution to expand its use. Additionally, its barrier performance could be enhanced by reaching the level of PLA matrix [8].

One of the great advantages of the development of biodegradable materials based on renewable resources is the reduction in plastic waste and, consequently, the reduction in environmental impact. In this sense, the study of the biodegradation process of these new biodegradable materials is of primary importance. Biodegradation is the degradation of the materials into environmentally acceptable products, such as water, carbon dioxide, and biomass, by the action of naturally available microorganisms, such as bacteria, fungi, and algae, under environmental conditions. In order to be meaningful, the biodegradation process must be linked to a duration comparable to the human scale, and specific biodegradation conditions. That is why it is more appropriate to define this type of material not only as biodegradable but also as compostable or biodegradable under composting conditions [9]. Thus, the term biodegradable describes a process, while the term compostable describes where and when the process will take place. If a material is compostable, it means that it is biologically degraded under aerobic conditions, producing carbon dioxide, water, inorganic compounds and biomass at the same rate as the rest of the organic matter that is being composted with, without leaving visible or toxic residues, turning into compost [10].

The enzymatic biodegradation of a material depends on the pH value, temperature, moisture, and also on its glass transition temperature and crystallinity degree [11,12,13,14]. The rate of enzymatic degradation decreases with crystallinity degree, crystal size, and glass transition temperature. In general, the process for rubbery materials is faster than that of glassy and/or highly crystalline ones.

The biodegradation process of PLA starts with a water absorption stage followed by hydrolysis and random cleavage of the ester bonds in the main chain of the polymer, thus a reduction in molar mass to oligomers and lactic acid is observed [11]. The outer carboxylic groups in the PLA molecules autocatalyze this process, which mainly occurs in the inner part of the polymer rather than on its surface [12]. The amorphous regions of the PLA polymer have a greater water absorption capacity since they undergo hydrolytic degradation before the crystalline regions [13]; this process leaves more space available for the undegraded chains to reorganize, thus increasing crystallinity and, consequently, rigidity and fragility of the material [14]. In a second stage, the low molar mass oligomers diffuse out of the polymer core and are naturally metabolized, depending on the biological environment to which the material is exposed. In applications where the product is composted at the end of its useful life, metabolization is carried out by microorganisms, providing carbon dioxide, water, and biomass as final products, thus closing the natural cycle of PLA [15]. In nature, the degradation of PLA is difficult under environmental conditions or in soil due to the greater resistance to the attack by microorganisms under mesophilic conditions (about 25 °C) [1]. A longer period under these conditions would increase the level of biodegradation, although it would imply lengthening the process for many months. For this reason, thermophilic conditions (about 58 °C) are necessary for industrial composting systems to rapidly reduce the molar mass of PLA without the need for catalysts, enabling the required levels of biodegradation that allow PLA to be considered as a compostable material [9].

PHB can be degraded by a wide variety of ubiquitous microorganisms in many ecosystems, both under aerobic and anaerobic conditions, without the formation of toxic products. These microorganisms are not necessarily accumulators of PHB, but rather use PHB as an exogenous carbon source [16]. The large size of the PHB individual macromolecules impedes their transportation through the cell membrane and, therefore, the microorganisms must be able to hydrolyze the PHB chains into their corresponding monomers (hydroxy acids) [16]. The microorganisms colonize the surface of the polymer, secreting depolymerases, which hydrolyze the PHB ester bonds, generating oligomers and subsequently monomers [17]. The latter are small enough to pass through the semi-permeable microbial membrane and metabolize into carbon dioxide and water under aerobic conditions [16]. Enzymatic degradation caused by microbes occurs on the polymer surface, causing significant surface erosion [18]. In addition, the hydrolytic degradation of PHB is generally a slow process that takes several months. This is due to the very high crystallinity and hydrophobic nature of the long pendant alkyl chains [19]. For this reason, a microbial environment is required for the degradation of PHB [15]. The main biodegradation mechanism of PHB is the enzymatic one, dissimilar to PLA, which does not biodegrade without a hydrolysis process previously occurring [11]. The degradation time depends on the nature of each polymer, as well as on the environmental conditions to which it is exposed. PHB degradation has been observed in mesophilic and thermophilic conditions and a large number of environments including aerobic, anaerobic, saline, and marine, among others [20].

This article aims to investigate the behavior of plasticized PLA/PHB blends by evaluating the role of composition and the factors affecting the kinetics of the disintegration under composting process. Even if the functional properties of plasticized PLA/PHB blends have been investigated by other authors [1,2,3,4,5,6,7], the evolution of their morphology, crystallinity, and thermal properties during composting tests have not yet been studied. The thermal, mechanical, morphological, and barrier properties of the materials used in this work have been extensively studied in our previous contributions [5,21,22]. In this contribution, changes in physical and thermal properties, due to the thermophilic degradation process under composting, have been monitored in light of the potential applications of the blends as packaging material. Moreover, the materials were subjected to the same temperature composting process, but in the absence of soil, with the intention of independently assessing the annealing effect of the samples. Besides, the overall migration of materials in different food simulants was also determined.

## 2. Materials and Methods

### 2.1. Materials

poly(lactic acid) (PLA 2003D, Mw = 236 kg mol^−1^, PDI = 3.3, 96 wt% l-isomer) was provided by NatureWorks^®^ (Minnetonka, MN, USA) and poly(3-hydroxybutyrate) (PHB, Mw = 492 kg mol^−1^, PDI = 6.3) was kindly supplied by PHB Industrial S.A. (Serrana, SP, Brazil). Tributyrin plasticizer (TB, 0.302 kg mol^−1^) and all of the chemicals were purchased from Sigma–Aldrich^®^(Merck KGaA, Darmstadt, Germany).

### 2.2. Films Preparation

PLA and PHB were blended in two weight ratios (70:30 and 60:40) and plasticized with TB (15 wt%), following previous results [21]. Masterbatches were prepared by using a Haake mixer at 185 °C with a rotation speed of 50 rpm for 3 min. PLA pellets were first introduced into the mixer, and once melted, PHB pellets and TB were also added. Each polymeric blend batch contained a total mass of 28 g. The melt-compounding procedure was conducted to obtain eight formulations, including the neat PLA and PHB homopolymers, PLA/PHB blends, and the corresponding plasticized materials. About 4 g of each masterbatch were then compression molded into ~150 μm films on a Delfabro press at 190 °C. Materials were kept between the hot plates for 1 min at 0.1 MPa until melting, and then for 3 min at 5 MPa. Finally, the films were fast-cooled at room temperature. The contents of each component in the final formulations are summarized in Table 1.

### 2.3. Size Exclusion Chromatography

Molecular weight was determined by size exclusion chromatography (SEC) at 25 °C, using an LKB-2249 instrument. A series of μ-Styragel columns, pore sizes 106, 105, 104, and 103 Å, were used with chloroform as an eluent (0.5 mL min^−1^ flow) of polymer solution (4–5 mg mL^−1^). The polymer was detected at 5.75 μm with a Miran 1A infrared spectrophotometer detector. A molecular weight calibration curve was obtained using poly(methyl methacrylate) as standard.

### 2.4. Overall Migration in Food Simulants

Overall migration tests were performed following the European Commission Regulation EU No. 10/2011 [23]. Two simulants were selected for analysis: ethanol 10% (*v/v*) (simulant A) and isooctane (simulant D1). Specifically, simulant A is designated for food products that have a hydrophilic character; instead, simulant D1 is designed to simulate the behavior of food products that have a lipid character. The test consisted of the total immersion of samples having a surface area of 25 cm^2^ in 25 mL of simulant. Films were kept in ethanol 10% (*v/v*) and isooctane for 10 days at 40 °C and 2 days at 20 °C, respectively, according to EU regulation No. 10/2011. After the incubation period, samples were removed and simulants were totally evaporated to obtain the residue, which was weighed in an analytical balance Mettler Toledo model AB (degree of accuracy of 0.1 mg). A blank test for each simulant without a sample was also run. The overall migration (OM) was calculated as the mass of migrant per dm^2^ of film surface, according to Equation (1).

OM = (M_s_ − M_b_)/A_s_ (1)

M_s_ is the mass residue obtained after evaporating the simulant that has been in contact with the sample, M_b_ is the mass residue of the blank test, and A_s_ is the surface area of the sample.

All of the tests were performed in triplicate and the OM was calculated as the average of three values. The legislation also provides an overall migration limit (OML) of 10 mg dm^−2^ [23].

### 2.5. Composting Tests

Composting degradation experiments of PLA and PHB-based materials have been performed by using mature compost kindly provided by HerAmbiente S.p.A. (Bologna, Italy) in a laboratory-scale test. Specimens of about 15 × 15 mm^2^ were weighted to obtain the initial mass and placed in a 100 mL bottle sandwiched between two layers of compost (20 g) moisten with deionized water (10 mL). Vessels were incubated in an SW22 Julabo water bath at 58 °C and 90% relative humidity, guarantying the aerobic conditions. At different incubation times, duplicate specimens were withdrawn, washed, and dried until constant weight. The percentage of gravimetric weight loss at each time was calculated as [(*m_i_* − *m_r_*)/*m_i_* · 100%], being *m_i_* and *m_r_* the initial and residual dry weight of samples, respectively. Photographs of specimens were taken for visual comparison during the different days in compost. Parallelly, specimens of each formulation were stored at 58 °C in absence of composting soil, to evaluate the effect of the temperature and humidity conditions separately from the bacteria degradation. These specimens were called ‘Annealed samples’, to differentiate them from the ‘Composted samples’ buried in compost and monitored at the same time intervals.

### 2.6. Surface Characterization

The microstructures of the PLA and PHB-based materials’ fractured surfaces were investigated through field emission scanning electron microscopy (FESEM, Supra 25-Zeiss, Dresden, Germany). Films were broken in liquid nitrogen and coated with a thin gold foil. The surface modifications of the film samples were also examined after composting tests.

### 2.7. DSC Measurements

Films were thermally characterized using differential scanning calorimetry (DSC) using a DCS6 Perkin-Elmer Instrument (Waltham, MA, USA) equipped with an intracooler set at −90 °C and under a nitrogen atmosphere (gas flow: 20 mL min^−1^). The samples were subjected to a first heating ramp from −70 to 200 °C at 20 °C min^−1^ (I scan), followed by an isothermal step of 3 min and a cooling ramp to −70 °C at 100 °C min^−1^, and finally to a second heating ramp from −70 °C to 200 °C at 20 °C min^−1^ (II scan). Incubated samples under annealing and composting conditions were subjected to a first DSC scan after different test times. Glass transition temperature (T_g_) was taken as the midpoint of the heat capacity increment ΔC_p_ associated with the glass-to-rubber transition. Crystallization temperature (T_c_) and melting temperature (T_m_) were determined as the maximum and minimum peaks of the crystallization exotherm and melting endotherm, respectively, with the corresponding Δ*H_c_* and Δ*H_m_* enthalpies calculated from the area under the peaks. For this purpose, the software OriginPro 8.5 was used. The degree of crystallinity (χ_c_) was evaluated as:

χ_c_ = [((Δ*H_m_* − Δ*H_c_*)/Δ*H_m_*_0_)/*ω* × 100%] (2)

Δ*H_m_* and Δ*H_c_* being the melting and crystallization enthalpy from DSC I scan, Δ*H_m_*_0_ is the melting enthalpy of PLA or PHB 100% crystalline (93 J g^−1^ and 146 J g^−1^, respectively) [24,25], and *ω* is the mass fraction of PLA or PHB in the sample.

### 2.8. TGA Measurements

The thermal stability of samples before and after incubation conditions was evaluated by thermogravimetric analysis (TGA) using a PerkinElmer TGA4000 Instrument (Waltham, MA, USA). The measurements were carried out under nitrogen atmosphere (gas flow: 40 mL/min) by heating from 30 to 700 °C at 10 °C min^−1^. The initial degradation temperature (T0) was calculated for a decomposition degree of 0.05 from TG curves, while the maximum degradation rate temperature (T_max_) was obtained from the first derivative TG curves (DTG) as the maximum peak value.

### 2.9. WAXS Analysis

Wide-angle X-ray scattering (WAXS) analysis of incubated samples was carried out at room temperature using a PANalytical X’Pert PRO diffractometer equipped with an XCelerator detector and a copper target (λ = 0.15418 nm). Data were acquired in the 5–60° 2θ interval, by collecting data for 100 s at each 0.10° step.

## 3. Results

### 3.1. Molecular, Thermal and Morphological Characterization

**Figure 1:**
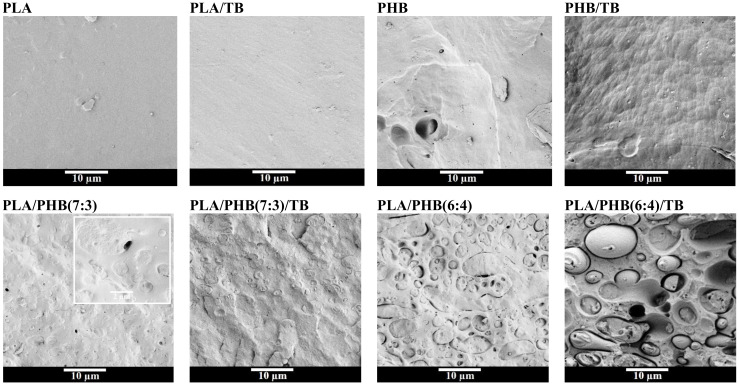
Morphological investigation of PLA and PHB based films fracture surface.

The effect of polymer blending and plasticization on the overall migration behavior and degradation rate of PLA and PHB-based products was assessed by analyzing the samples under food simulants and composting conditions for several days. The properties of the materials prior to any experiment are presented in Table 2 and Table 3, and FESEM investigation of their fractured surfaces is illustrated in Figure 1. The molecular weight of PLA and PHB polymers before and after melt-processing and compression-molding into thin neat/plasticized films have been determined by SEC analysis (Table 2). As expected, samples showed a significant decrease in molecular weight (M_w_) compared to the received commercial raw material, and a further reduction in M_w_ after plasticization was also observed. These results are associated with the thermal degradation by chain-scission that typically occurs during high-temperature processing. Accordingly, this effect was more noticeable for PHB (about 37% molecular-weight reduction), which has a lower thermal stability than PLA [1,26].

The values for thermal transitions, estimated by differential scanning calorimetry tests (Table 2 and Table 3), revealed the amorphous (χ_c_ around 1%) and glassy (T_g_ around 45–62 °C) nature of PLA and PLA/TB films, and the highly crystalline (χ_c_ around 51–58%) and rubbery (T_g_ around −14 to 1 °C) state of PHB and PHB/TB films. PLA/PHB and PLA/PHB/TB blends proved to be biphasic materials with double thermal behavior characteristic of each PLA-rich and PHB-rich phases. The reduced proportion of crystalline PHB used to prepare the blends, which turned out to be effective as a nucleating agent for PLA, induced a considerable increase in its crystallinity degree.

Cross-section morphology (Figure 1) of PLA and PLA/TB samples resulted to be smooth and uniform, according to their amorphous and brittle nature. PHB and PHB/TB samples presented irregular fracture surfaces, due to their crystalline structure. All of the PLA/PHB blends consisted of two phases, in agreement with thermal investigation. It was observed that PHB particles in the PLA/PHB(7:3) and PLA/PHB(7:3)/TB blends are well dispersed in the form of small sphere-shaped domains in the PLA matrix. On the other hand, PHB particles in the PLA/PHB(6:4) and PLA/PHB(6:4)/TB blends appear to be arranged as large ellipsoids, which could indicate the creation of a co-continuous morphology formation. Moreover, the blend formulations containing less PHB content seem to present a better interfacial adhesion between PLA and PHB phases.

### 3.2. Overall Migration

**Figure 2:**
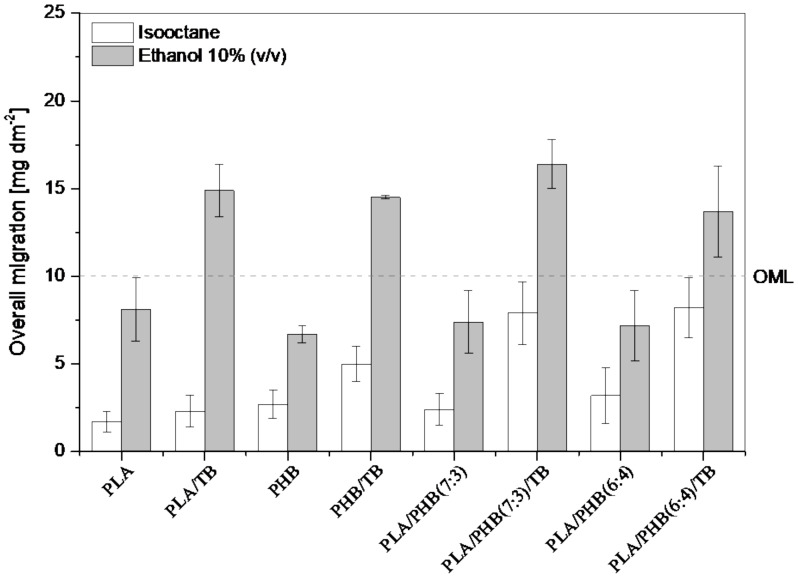
Overall migration data in isooctane and ethanol 10% (*v/v*) for PLA and PHB-based films. The dashed line indicates the overall migration limit (OML).

Overall migration (OM) tests were carried out by selecting two simulants for the evaluation of the total amount of non-volatile substances that might migrate from the PLA and PHB-based films into the foodstuff (or simulant). Figure 2 shows the overall migration values for the different samples and simulants (A, ethanol 10%, and D1-isooctane). The overall migration limit (OML) of 10 mg dm^−2^, as stated by the European Regulation [23], is indicated on the graphic. The total migration behavior of the different materials followed the same trend in both simulants (A or D1): the migration levels increased for all samples containing the TB plasticizer, compared with their unplasticized counterparts. This could be explained by the fact that the addition of TB increased the low-molar mass compounds’ mobility through the polymer matrix [2], in agreement with the decrease in glass transition temperatures (Table 2 and Table 3). Lower migration levels were found in the non-polar solvent, i.e., isooctane. This behavior is in accordance with the results obtained by other researchers that studied the OM of PLA based [27,28] and PHB or PHBV based films [29,30]. Moreover, values of migration in D1 were below the OML for each formulation. Plasticized films (PLA/TB, PHB/TB, PLA/PHB(7:3)/TB and PLA/PHB(6:4)/TB) exceeded the OML in the aqueous polar simulant (A) after 10 days of incubation at 40 °C, while migration levels of unplasticized samples (PLA, PHB, PLA/PHB(7:3) and PLA/PHB(6:4)) were found to be within the European legislation limit. Thermodynamic properties, such as polarity and solubility, are the main factors that control the migration process, due to interactions between polymers, migrants, and food simulants [2,27]. For instance, if a migrant has good solubility in the simulant, it would be released into the foodstuff rather than remain in the polymer matrix. Temperature is also an extremely important factor; the higher the temperature, the higher the flexibility of the polymer molecules, and, therefore, the higher the migration rates [31]. It was reported that polar solvents, such as ethanol aqueous solution, were aggressive to PLA and PHB systems due to polymer hydrolysis [28,32]. Acidic organic compounds, such as crotonic and lactic acids, and linear and cyclic oligomers (reported to be the end products of PHB and PLA degradation processes [7,33,34]), are polar and, therefore, soluble in water [35]. In this sense, most of the residue found in the polar solvent (A) could be mainly attributed to the migration of water-soluble degradation products of PLA and PHB formed by hydrolytic degradation during the OM tests [28,36,37] and/or by partial thermal degradation of the polyesters during the films’ preparation process under elevated temperature [3]. This last assumption is in good agreement with the reduction in the weight-average molar mass of polymers after the melt-mixing and compression molding film preparation, as shown in Table 2. Another important factor to consider is the possible migration of the hydrophobic plasticizer despite its low water solubility, caused by structural changes that may see the materials suffer under the high-temperature test condition (40 °C for 10 days) [36]. Furthermore, the good solubility of TB in alcohol is understood [38]. The combination of all of these factors, along with the extreme environmental conditions of the tests, leads the OML to exceed the limit for the plasticized formulation films in polar systems. On the other hand, after 2 days of incubation at 20 °C in isooctane (D1), all of the studied materials showed global migration values below OML, indicating that the material properties comply with the EU legislation for fatty food contact materials. The maximum migration level was detected for PLA/PHB(7:3)/TB and PLA/PHB(6:4)/TB formulations at approximately 8 mg dm^−2^. The migration phenomenon of the non-polar materials in the non-polar simulant (D1) could be explained by the affinity of the polymers with the solvent. This could lead to the swelling of the polymer matrix due to the absorption of the liquid simulant, increasing the free volume and, consequently, enhancing the migration of small molecules [31]. Furthermore, in this case, the test environmental conditions were less aggressive, so it could be assumed that a structural modification of materials might not occur.

### 3.3. Disintegration Tests under Composting Conditions

The disintegration level of PLA and PHB blends was tested by compost degradation experiments under controlled aerobic conditions. Each sample recovered at different incubation times was analyzed according to the following parameters: visual modifications (such as color, texture, and dimensions), gravimetric weight loss or degree of disintegration, thermal and structural properties. Composting is a natural process that turns organic material into a rich soil, called compost or humus, by the action of microorganisms that break down the organic matter and produce carbon dioxide, water, and heat [39]. As outlined in the literature review, PLA and PHB biodegradation mechanisms are rather different. PHB degradation is essentially enzymatic; exoenzymes from bacteria and fungi present in the composting soil break down the high molecular-weight chains yielding smaller ones able to pass through the semi-permeable external bacterial membranes, and thus to be metabolized. The enzymatic degradation caused by microbes mainly occurs at the polymer surface due to the large size of the enzyme, causing surface erosion [15]. On the other hand, PLA degradation undergoes a two-step process. During the first two-week stage, bulk water diffusion produces non-enzymatic hydrolysis of PLA, reducing its molecular weight by random chain scissions of the ester groups, and causing a cross-section erosion [15,39]. When PLA molecular weight reaches about 10,000–20,000 g mol^−1^, microorganisms start to assimilate the oligomers and lactic acid on a second degradation stage [40]. The biodegradation rate depends on many factors that include environmental conditions, such as temperature or moisture level, polymer characteristics, such as composition, mobility, crystallinity, molecular weight, and presence of additives, among others [15,39,41]. These features were evaluated in the laboratory-scale composting tests.

#### 3.3.1. Changes of Macroscopic Appearance

**Figure 3:**
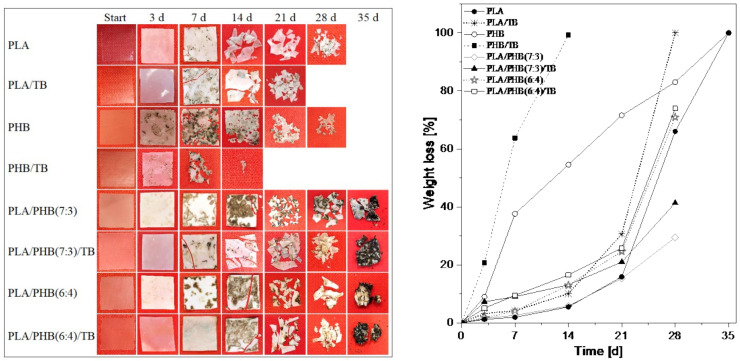
Visual appearance (**left**) and gravimetric weight loss % (**right**) for PLA and PHB based materials at different stages of incubation in compost.

The visual appearances of PLA and PHB-based materials after different times under composting conditions are shown in Figure 3 (left), where it is possible to verify the biodegradable character of the studied formulations. Changes in color and haze of all samples were observed after only three days of incubation, and this effect was accentuated on the seventh day. The increase in opacity is directly related to alterations in the mechanisms of light diffusion through the samples, due to structural changes caused by water absorption, hydrolytic degradation [42], crystallinity increase (which was later evidenced by DSC and WAXS analysis), and pore formation during the degradation process [43].

It is known that these phenomena mainly take place in the amorphous region of the polymer structure, thus it was observed that the whitening of films was higher for the amorphous material, i.e., neat PLA and samples containing PLA amounts higher than 50 wt%. The progressive loss of the samples’ structural integrity led to a reduction in thickness, increase in surface roughness, formation of superficial cracks, visual fragmentation, and fragility of the materials, especially after 14 days of composting for PLA and PLA/PHB blends-based materials. On the other side, PHB and PHB/TB films did not show evident signs of embrittlement at any incubation stage. This result is particularly interesting, considering that PHB is highly crystalline. In this case, a gradual appearance of macroscopic holes on films was observed, followed by a size decrease in samples, through fragmentation, until total disintegration. This aligns with the degradation mechanism by surface erosion proposed for PHB, while bulk erosion seems to have caused the fragmentation of PLA matrix samples. Similar results were found by other authors [44].

The detection of visual changes can be used as the first indication of any hydrolytic process and microbial attack [15]. To obtain further information about the degradation mechanism, more sophisticated observations have been made using scanning electron microscopy (FESEM) as discussed later.

#### 3.3.2. Weight Loss Measurements

The evolution of the gravimetric weight loss of samples buried in compost was evaluated and the results are shown in Figure 3 (right). The analysis of the disintegration of neat polymers revealed that PHB degraded faster than PLA since the beginning of the test; after 14 days, PLA sample lost only 5% of its initial mass, while PHB sample reached 55% of weight loss. It was reported that PLA biodegradation starts with non-enzymatic hydrolysis by a random cleavage of the polymer chains and this effect usually takes place during the first two weeks of compost incubation [15]. This mechanism is especially common for high M_w_ PLA [45]. The hydrolysis process is affected by the rate of water diffusion through the polymer and, therefore, by the mobility of the polymer chains, which is reflected in its glass-to-rubber transition temperature. PLA displayed a T_g_ around 62 °C (Table 2), which is very close to the test temperature (58 °C). In such conditions, the polymer could still be in a glassy state, or at least the chain mobilization is limited, slowing down the hydrolysis process. Furthermore, the gravimetric weight loss would be considerable once the enzymatic degradation of the polymer occurs, i.e., once the microorganisms metabolize the organic compounds. In this sense, Fabbri et al. [46] reported a substantial decrease in the molecular weight of PLA during a hydrolytic degradation experiment, confirming the beginning of the degradation process, without registering any gravimetric weight loss. These factors would explain the low disintegration rate of PLA during the first weeks of incubation. Nonetheless, after 21 days of the experiment, PLA disintegration speeded up. On the other hand, the faster biodegradation rate of PHB could be explained as a result of the great mass loss caused by enzymatic surface erosion. Moreover, the low T_g_ value around 1 °C (Table 2) would facilitate the accessibility to the rubbery amorphous polymer chains, present on the film surface, to the extracellular enzymes of the microorganisms present in the compost, despite the presence of a consistent crystalline high-packed phase. Finally, after 35 days of incubation, no sample residues of PLA and PHB were found in the compost, which would indicate the complete degradation of both homopolymers after 5 weeks of incubation.

Plasticized PLA and PHB films showed a higher disintegration rate than their unplasticized counterparts. It should be considered that PLA/TB and PHB/TB presented lower M_w_ values than neat samples (Table 2) and, in general, polymers with lower molecular weight should degrade faster than longer macromolecules [15]. Furthermore, the small and high-mobile TB molecules may be easily released into the composting medium, where they are preferentially attacked by microorganisms and, in this way, the early degradation of these molecules would interfere with the structural integrity of the polymers’ matrix, increasing considerably the surface area for enzymatic and/or hydrolytic attack [15]. The thermal properties data of the composted samples, discussed later in this article, bore out the loss of plasticizer in the test initial stages. Moreover, in the first process step during the plasticizer release, hydrolysis may also occur, producing acid groups which would promote the hydrolysis of polymer chains [47,48] through an autocatalytic effect and, consequently, accelerate the disintegration process. The incorporation of plasticizer also reduced the polymers’ glass transition temperatures, thus increasing the molecular chain mobility [5,21] and the disintegration phenomenon rate. PLA/TB presented a T_g_ around 46 °C, while the one of PHB/TB was about −14 °C (Table 2), being both rather lower than the assay temperature. The PHB/TB sample was completely disintegrated after 14 days of testing, whereas the PLA/TB film after 28 days, reducing considerably the composting times compared to the neat homopolymers.

Biodegradation of PLA/PHB blends is expected to start with a combined degradation mechanism (enzymatic and non-enzymatic hydrolysis). However, it is worth noting that the predominant polymer in all blend formulations is PLA, hence degradation might have been triggered by bulk water hydrolysis. PLA/PHB(7:3) sample showed almost the same weight loss percentage of neat PLA up to 21 days, while the slight increase in PHB content in the PLA/PHB(6:4) sample speeded up the process.

This could be explained by considering the differences in morphology of both formulations observed by FESEM (Figure 1). In the PLA/PHB(7:3) blend, PHB is dispersed as much smaller domains in the PLA continuous phase, than in PLA/PHB(6:4), where it is dispersed as large ellipsoids, indicating that a co-continuous morphology was starting to form. Therefore, in the second case, the enzymes would have easier access to degrade the PHB polymer chains, accelerating the process. Once again, the incorporation of TB increased the biodegradation rate of PLA/PHB(7:3)/TB and PLA/PHB(6:4)/TB samples. It should be noted that the weight measurements of PLA/PHB and PLA/PHB/TB blends were taken until day 28, due to the high degree of disintegration of the materials and the presence of attached soil and fungi, which made it difficult to recover the piece samples from the compost (Figure 3 (left)-35d). After 42 days of incubation, no traces of PLA/PHB blends samples were found in the compost.

#### 3.3.3. Surface Morphological Analysis

**Figure 4:**
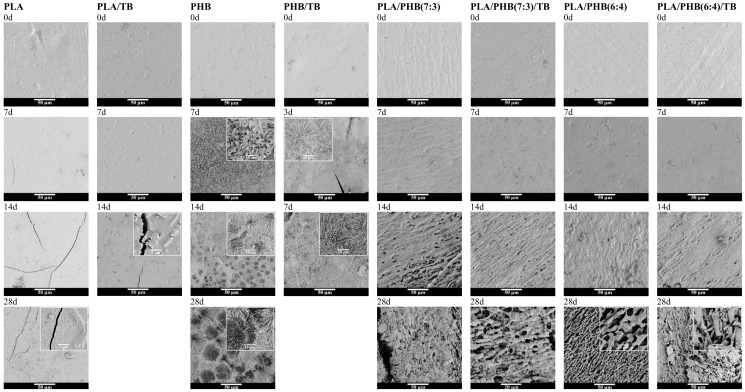
FESEM images of PLA and PHB-based materials surfaces after different stages of incubation in compost at 58 °C.

Information about the degradation mechanism can also be obtained from the surface topography of the samples. The films’ surface changes during the biodegradation process were investigated by scanning electron microscopy. Figure 4 shows the surface morphology of films after different days of incubation in compost. All of the films exhibited smooth and homogeneous surfaces prior to the experiment.

PLA and PLA/TB films showed cracked surfaces with deep fractures and holes that increase in number with the incubation days [47,49]. These types of topographies are typical of hydrolytic degradation mechanisms, where the cleavage process of polymer chains occurs mainly inside the matrix (cross-sectional area) rather than on the surface [12,50]. External cracks may have formed during the water diffusion into the polymer matrix amorphous regions and during the migration of low molecular-weight compounds into the composting medium. From the PLA and PLA/TB higher magnification micrographs, it is also possible to observe rough and porous surfaces and the presence of residual bacteria. PHB and PHB/TB micrographs revealed a significant surface erosion of the samples as a result of bacterial consumption, which would corroborate the hypothesis of a mainly enzymatic degradation mechanism [44,51]. Crystalline spherulites were identified on the films’ surface. This can be explained by a preferential degradation of the amorphous portion of the polymer, exposing the slower degrading crystalline parts. Rather large spherulites, grain boundaries, and triple junctions can be fully seen on PHB degraded surfaces after 28 days of incubation in compost.

Other authors have also observed these differences between the degradation mechanisms of PLA and PHB through electron microscopy. Doi et al. [52] presented SEM images of enzymatically degraded PHB with an irregular and highly eroded surface, while its cross-section showed no signs of degradation. Zhang et al. [1] observed the fractured surface of degraded PLA/PHB blends in compost and concluded that PHB was attacked from the sample surface, but PLA degradation took place throughout the whole of the sample. Materials based on PLA/PHB blend showed a topography evolution over degradation time, rather different from homopolymers, which suggests a combined degradation mechanism. After 2 weeks of incubation in compost, surface erosion of all the samples was indeed noticeable and many cavities and deep holes were found. When the degradation time was increased to 28 days, it was seen that the erosion had spread gradually to the interior of the matrix and a network of cavities was formed for all systems. On the high-resolution micrographs, it is interesting to observe that the remaining phase supporting the structural integrity of film samples is essentially crystalline, confirming again the preferential degradation of the amorphous fraction of the matrix. Therefore, it could be said that the PLA/PHB-based materials suffered both surface and massive (volume) erosion due to microbial and hydrolytic attacks.

#### 3.3.4. Thermal and Structural Analysis. Comparison with Annealed Samples

Composted samples and the corresponding blanks (i.e., specimens annealed at 58 °C) were studied by DSC, TGA, and WAXS, to assess and compare the thermal and structural modifications under both conditions at specific time intervals. Furthermore, changes in molecular weight due to composting of PLA and PLA/TB samples were assessed (Table 4).

**Figure 5:**
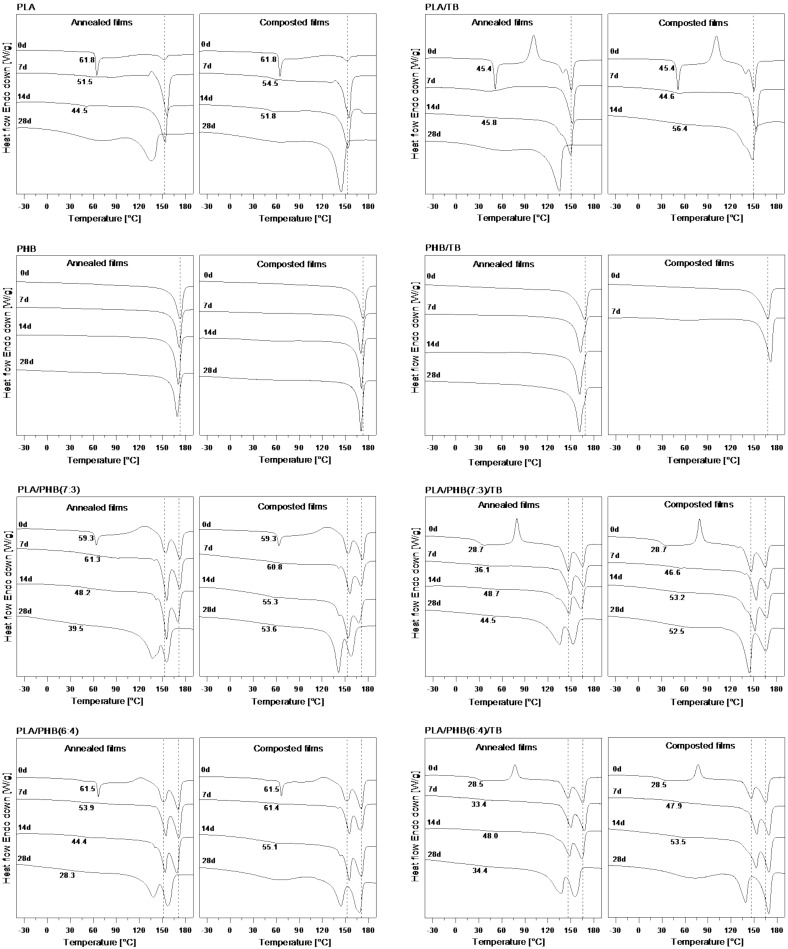
DSC first scans of PLA, PLA/TB, PHB, PHB/TB, PLA/PHB(7:3), PLA/PHB(7:3)/TB, PLA/PHB(6:4) and PLA/PHB(6:4)/TB films under annealing (**left**) and composting (**right**) conditions after 0, 7, 14, and 28 days. The numbers at each curve represent T_g_ (°C) values of PLA. Heating rate: 20 °C min^−1^ under nitrogen flow.

In Figure 5, the calorimetric traces of annealed and composted samples at different days of incubation are reported, while the thermal parameters are summarized in Table 5. DSC curve of the as-prepared compression-molded PLA film (time 0) showed a clear heat flow shift associated with the T_g_, superimposed with a typical sharp endothermic peak, followed by a flat exothermic crystallization region and a small endothermic melting peak. Moreover, Δ*H_c_* ≈ Δ*H_m_*, indicating the completely amorphous state of the material. Annealed and composted PLA films showed a similar evolution of thermal behavior within the first two weeks of incubation. A decrease in the T_g_ values was registered, to a higher extent in the annealed samples, with the T_g_ steps becoming less pronounced but still visible until day 14. Furthermore, the crystallization peak disappeared, and the heat of melting grew significantly with the incubation time. These features were attributed to the polymer hydrolysis process, during which a random cleavage of polymer chains occurred, consequently decreasing the molecular weight, and increasing polydispersity index of PLA (Table 4) [53]. When the polymer chain length decreases, the shorter macromolecule chains have greater mobility and require less energy (lower T_g_) to start moving [54]. Simultaneously, an increase in mobility enables a secondary crystallization of the amorphous zones, which is reflected in an increase of the heat of melting and, therefore, of the crystallinity index of annealed and composted films. It is interesting to note a slight increase in T_m_ on the first days for the neat film (time 0) and its subsequent decrease after the second week.

**Figure 6:**
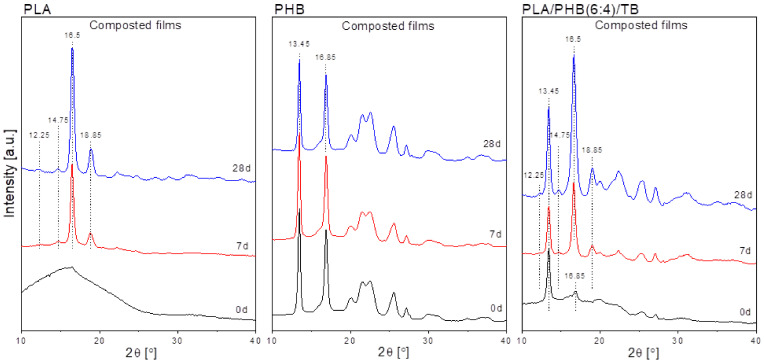
WAXS spectra of: PLA (**left**), PHB (**center**) and PLA/PHB(6:4)/TB (**right**) films incubated in compost for 0, 7 and 28 days.

At the first stage, the thermal annealing produces a reorganization of the crystalline phase that melts at a higher T_m_ but as the hydrolysis process proceeds, the formation of less perfect crystalline structures, that melt at lower temperature and in a less homogeneous way (i.e., the peak is broader). After four weeks (time 28d) of storage, the melting peak of annealed PLA films shifted to a lower temperature (around 17 °C) and became notoriously wider. This behavior was less noticeable in the composted PLA film (melting peak decreased around 8 °C) since the shortest chains produced by water hydrolysis are those preferentially attacked by microorganisms. Moreover, the samples exposed to composting conditions suffered both annealing recrystallization and bacteria degradation of the polymeric amorphous phase fraction, which explains the higher increment in endotherm melting areas (around 83-fold for composted film compared with 53-fold for the annealed film) and the strong embrittlement and fragmentation of composted samples (Figure 3-left). The promotion of the crystalline phase has also been detected by WAXS analysis (Figure 6). Finally, the great similarity between the DSC curves of the annealed and composted materials during the first two weeks of incubation and the subsequent notable change in their thermal behavior after the third week would confirm that the PLA degradation mechanism starts mainly with a non-enzymatic hydrolysis process.

Annealed and composted PLA/TB films also exhibited signs of hydrolytic degradation with testing time, such as molecular weight decrease (Table 4), the disappearance of the crystallization exotherm peak from their calorimetric curves, and the shift of T_m_ and increment in the melting heat. However, contrary to what was above reported for neat PLA films, an increase in T_g_ values was registered, especially for those PLA/TB samples exposed to composting conditions. This could be explained by plasticizer exudation and/or its early degradation, which reduced the plasticizing effect. The heating scans of the annealed PHB samples showed marginal thermal-data changes compared with time-0 film. Even though all polyesters are susceptible to degradation by hydrolysis to some extent [55], the high degree of polymerization, high crystallinity, isotacticity, and hydrophobicity of PHB are some of the factors that limit the hydrolysis of the polymer [18], making its hydrolytic degradation less evident. The same behavior was found for the composted samples, in agreement with the proposed superficial degradation mechanism of PHB, in which the bulk polymer matrix is not affected as much. Moreover, the degree of crystallinity of the polymer, reflected in the endotherm underlying area (Δ*H_m_*), remained practically unchanged under both conditions (increments of only 9 and 2% were registered for Δ*H_m_* at time 28d). This was as well revealed by WAXS (Figure 6), in line with the negligible embrittlement variation of composted PHB samples (Figure 3 left).

However, it is possible to accelerate the hydrolytic degradation of PHB by adding plasticizers or other polymers easily hydrolyzable, which disturb the PHB high crystallinity [19]. Concerning the PHB/TB annealed samples after 28 days, a higher reduction (around 4.2%) in melting temperatures was identified when compared with the unplasticized PHB sample (around 2.1%), indicating a higher hydrolytic degradation degree. This would coincide with the PHB/TB highest disintegration rate. Two melting peaks were observed in the DSC curves of PLA/PHB and PLA/PHB/TB blends, corresponding to PLA and PHB phases, respectively. Δ*H_m_* of each polymer was obtained using a Gaussian multi-peak mathematical deconvolution technique and T_m_ temperatures were designated as the minimum values of those peaks. An even greater extent of hydrolytic degradation of PHB was detected when blended with PLA, evidenced by a further decrease in T_m_ values for PHB in the annealed blends (around 10% for PLA/PHB(7:3), 8% for PLA/PHB(6:4), 7.6% for PLA/PHB(7:3)/TB and 6.4% for PLA/PHB(6:4)/TB). T_m_ of both polymers decreased, while only PLA heat of melting considerably increased with testing time, for both annealed and composted materials, indicating an increase in its crystallinity degree. PHB-phase melting enthalpy remained almost invariant. The increment in crystallinity in blends may suggest that the PLA chains in the amorphous region of the composted blend samples residues are more restricted by the crystalline regions of both PLA and PHB domains and, therefore, are more hydrolysis-resistant [56], which could explain the longer time required to completely disintegrate PLA/PHB and PLA/PHB/TB blends (Figure 3, right). Concerning the PLA-phase glass transition behavior in blends, the T_g_ values decreased over time for the unplasticized blends, while they increased for the plasticized ones, indicating the loss of TB.

The samples were also studied by wide-angle X-ray scattering. As an example, in Figure 6 the WAXS patterns of composted PLA, PHB, and PLA/PHB(6:4)/TB films incubated for 0, 7, and 28 days are reported. The degree of crystallinity of PLA significantly increased with composting time. The starting sample (time 0) showed the amorphous halo characteristic of its amorphous nature, in agreement with the calorimetric results (Table 2). After 7 days, the marked reduction in the amorphous halo and the presence of two strong peaks at 2θ = 16.5° and 18.85° and two weaker peaks at 2θ = 12.25° and 14.75°, confirmed that poly(lactide) crystalline structures were formed [57], in line with the DSC results and the transparency loss of composted samples. These peaks gained intensity after 28 days, revealing an even greater increment in the crystalline/amorphous ratio. PHB patterns did not show apparent changes with composting days, indicating the negligible crystallinity degree variation, as confirmed by DSC.

Typical PHB crystalline diffraction peaks at 2θ = 13.45° and 16.85° were found [6]. WAXS pattern of PLA/PHB(6:4)/TB starting sample (time 0) revealed an amorphous halo with two strong peaks at 2θ = 13.45° and 16.85°, corresponding to PHB-phase. After 7 and 28 days of composting, new diffraction peaks at 2θ = 12.25°, 14.75°, and 18.85° appeared, corresponding to the PLA-crystalline phase. Moreover, the peak at 2θ = 16.85° (PHB-phase) shifted to 16.5° (PLA-phase), intensified and became wider, indicating the overlapping of both peaks. Finally, the intensity of 2θ = 13.45° (PHB-phase) slightly increased with time, according to DSC data.

**Figure 7:**
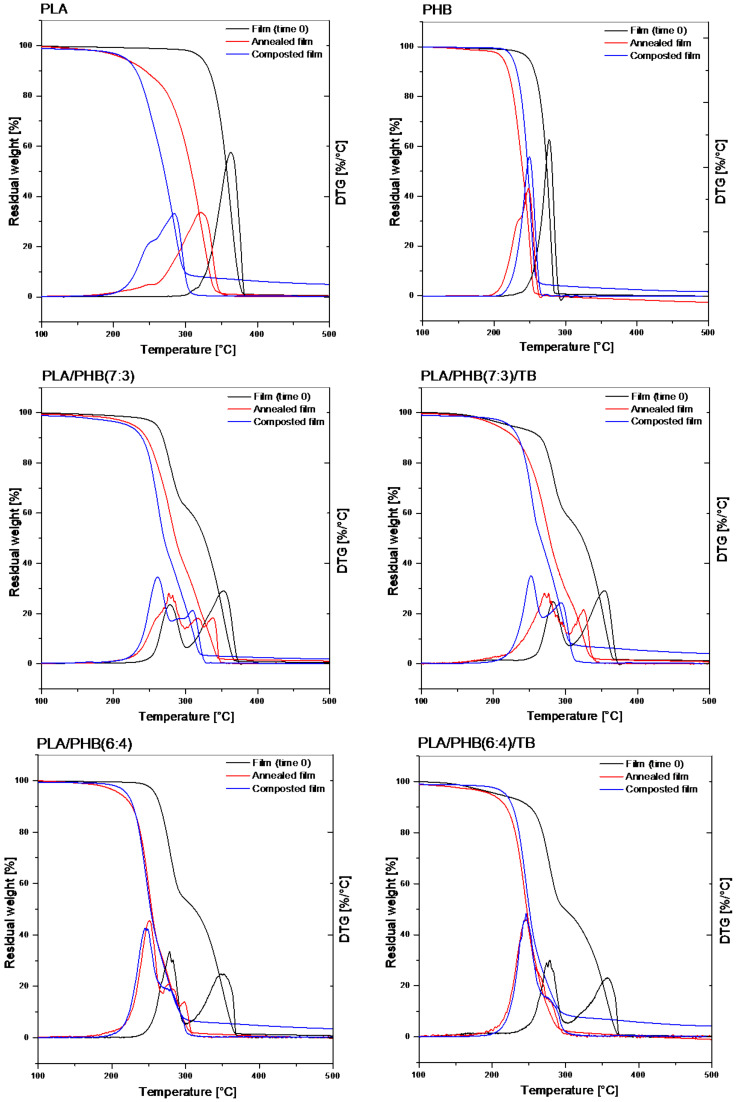
TG and DTG traces of PLA, PHB, PLA/PHB(7:3), PLA/PHB(7:3)/TB, PLA/PHB(6:4), and PLA/PHB(6:4)/TB samples of neat films (black line) and under annealing (red line) and composting (blue line) conditions after 28 days of incubation.

The thermal stability of annealed and composted samples was investigated by TGA before and after 28 days of incubation. The analysis was performed for those formulations characterized by sample residues after 4 weeks of incubation in compost, i.e., PLA, PHB, and PLA/PHB-PLA/PHB/TB blends. Thermogravimetric (TG) and derivative thermogravimetric (DTG) data are reported in Figure 7 and Table 6.

PLA and PHB samples suffered a single-step thermal degradation, PLA being more thermally resistant than PHB, while PLA/PHB blends were degraded into two steps, each one corresponding to the degradation of the individual polymers. The blending of the two polymers reduced (about 5–12 °C) the PLA maximum degradation temperature (T_max_), and slightly increased (about 1–6 °C) that one for PHB. Furthermore, the plasticization of blends compromised the initial thermal stability of PLA/PHB/TB formulations, reducing (about 38–50 °C) their initial degradation temperature (T0) when compared with unplasticized blends. This can be explained by the presence of TB, which degrades at lower temperatures, and it is consistent with the faster composting disintegration rate found for the plasticized films. Both degradation temperatures decreased with annealing and composting time. In general, the reduction in T0 was larger for the annealed samples, since the low-molecular weight chains produced by hydrolysis degrade at lower temperatures. Under composting conditions, these chains are preferentially attacked and eliminated by bacteria. While a slight decrease (around 10–15%) was observed for T0 of neat PHB after 28 days, confirming the low change of PHB structural integrity, neat PLA films showed the highest reductions (around 33–34%). Unplasticized blends showed intermediate reduction values (around 13–16% in PLA/PHB(7:3) and 15–18% in PLA/PHB(6:4)). Notably, the T0 of plasticized blends did not change, or it even was raised, for PLA/PHB(7:3)/TB and PLA/PHB(6:4)/TB composted formulations after 28 days, evidencing the plasticizer early degradation, as already evidenced by the DSC analysis.

The reduction in T_max_ was greater for the composted samples than for the annealed ones, a fact attributed to the synergic effect of hydrolytic and enzymatic degradation. As a matter of fact, PLA presented the highest percentage reductions due to the more aggressive hydrolytic attack, which it undergoes. After 28 days, the reduction in T_max_ in PLA component did not show large differences between PLA, PLA/PHB(6:4) and PLA/PHB(6:4)/TB blends (around 21–23%, corresponding to 74–82 °C), accordingly with their similar disintegration weight loss degree after 28 days (Figure 3). On the other hand, the reductions of T_max_ corresponding to the PHB thermal degradation only reached 10–12% after 28 days in all formulations. The lowest reductions (around 0–6%) were registered for PHB in PLA/PHB(7:3) blend, which could be explained by the material morphology of small, dispersed, and difficult-to-attack PHB domains in the PLA matrix (Figure 1).

## 4. Conclusions

Fully natural and bio-based PLA-PHB blends were investigated based on their overall migration behavior under food simulants and their biodegradation in thermophilic composting conditions. The total migration values of all film formulations immersed in a fatty (isooctane) food simulant were below the limit indicated in the current legislation. However, plasticized films containing 15 wt% of tributyrin were determined as inappropriate aqueous (ethanol 10% (*v/v*)) food contact materials. The synergic effect of the polymer hydrolysis process and the plasticizing effect of TB, which increases the free volume and chain mobility, caused the OML to exceed in the polar simulant. The thermal annealing of samples in absence of compost but at the same composting temperature revealed that PLA undergoes a much more aggressive hydrolysis process than PHB and that the hydrolytic degradation of the latter can be accelerated by blending with TB or PLA. PHB presented higher susceptibility towards disintegration in composting than PLA, losing gravimetric weight at a faster rate. The degradation process was indeed accelerated with the incorporation of plasticizer. Blending PLA with a PHB content of around 25–40 wt%, enhanced the degradation rate of PLA during the first three weeks, but it prolonged the total disintegration of one week compared to pure PLA. It was observed that PLA significantly increased its crystalline content in all samples containing a PLA amount higher than 50 wt% during the composting test, due to the erosion and bacterial attack of the amorphous parts. Finally, the study underlined the successful and tunable compostability of PLA-PHB-based materials as a sustainable end-life option and their suitability for prospective application as fatty food packaging material.

## Figures and Tables

**Table 1:** Compositions of the obtained materials.

	PLA (wt%)	PHB (wt%)	TB (wt%)
PLA	100	/	/
PLA/TB	85	/	15
PHB	/	100	/
PHB/TB	/	85	15
PLA/PHB(7:3)	70	30	/
PLA/PHB(7:3)/TB	59.5	25.5	15
PLA/PHB(6:4)	60	40	/
PLA/PHB(6:4)/TB	51	34	15

**Table 2:** Molecular and thermal characterization data of neat and plasticized PLA and PHB.

Samples	M_w_^a^ (g/mol)	Reduction in M_w_ (%)	PDI ^a^	T_g_^b^ (°C)	T_c_^b^ (°C)	T_m_^b^ (°C)	χ_c_ ^b^ (%)
PLA *	235,700	/	3.3				
PLA	208,050	12	3.3	61.8	124.0	152.1	0.9
PLA/TB	165,600	30	6.1	45.4	101.1	150.6	0.6
PHB *	492,330	/	6.3				
PHB	308,650	37	3.9	1.1 **	/	172.9	58.4
PHB/TB	277,500	44	4.4	−14.5 **	/	168.1	51.5

^a^ By SEC. ^b^ By DSC I scan. * Commercial polymer in pellets as received. ** T_g,PHB_ was obtained from DSC II scan.

**Table 3:** Thermal characterization data of neat and plasticized PLA/PHB blends.

Samples	T_g,PHB_^b^ (°C)	T_g,PLA_^a^ (°C)	T_c,PLA_^a^ (°C)	T_m,PLA_^a^ (°C)	T_m,PHB_^a^ (°C)	χ_c,PLA_^a^ (%)	χ_c,PHB_^a^ (%)
PLA/PHB(7:3)	−0.8	59.3	126.3	153.6	172.6	6.6	41.1
PLA/PHB(7:3)/TB	−19.6	28.7	79.2	146.6	165.0	11.8	51.9
PLA/PHB(6:4)	0.7	61.5	121.5	151.8	170.7	28.8	46.8
PLA/PHB(6:4)/TB	−17.9	28.5	76.9	146.4	165.3	20.7	47.2

^a^ By DSC I scan. ^b^ By DSC II scan.

**Table 4:** Changes in molecular weight (M_w_) and polydispersity index (PDI) due to composting of PLA and PLA/TB films.

	PLA			PLA/TB		
Time (d)	0	14	21	0	14	21
M_w_ (g/mol)	208,050	13,300	10,500	165,600	11,000	10,900
PDI	3.3	1.08	1.05	6.1	1.08	1.05

**Table 5:** Thermal properties obtained during the first DSC scan of annealed (A) and composted (C) films of neat and plasticized PLA, PHB, and PLA/PHB blends at different test times. (n.d.: not detected; for these samples, T_g_ could not be detected in the DSC thermograms).

	Time (d)	T_g,PLA_ (°C)		T_m,PLA_ (°C)		ΔH_tot,PLA_ * (J/g)		T_m,PHB_ (°C)		ΔH_tot,PHB_ * (J/g)	
		a	c	a	c	a	c	a	c	a	c
PLA	0	61.8		152.1		0.8					
	7	51.5	54.5	156.1	154.6	32.4	37.5				
	14	44.5	51.8	153.7	153.5	39.7	39.8				
	28	n.d.	n.d.	135.4	144.2	42.4	66.2				
PLA/TB	0	45.4		150.6		0.5					
	7	n.d.	44.6	151.9	153.5	32.5	35.8				
	14	45.8	56.4	149.3	148.3	40.8	55.4				
	28	n.d.	/	134.9	/	57.6	/				
PHB	0							172.9		85.3	
	7							171.7	169.9	81.8	82.4
	14							170.7	170.8	100.8	100.9
	28							169.3	170.2	92.9	87.0
PHB/TB	0							168.1		63.9	
	7							162.4	171.4	66.2	78.1
	14							161.5	/	72.8	/
	28							161.1	/	79.7	/
PLA/PHB(7:3)	0	59.3		153.6		4.3		172.6		17.9	
	7	61.3	60.8	155.8	156.4	30.1	24.5	172.5	170.8	20.5	16.7
	14	48.2	55.3	155.4	154.2	35.9	36.7	170.1	167.9	20.8	17.0
	28	39.5	53.6	137.1	141.8	45.6	35.5	155.4	157.8	25.6	18.9
PLA/PHB(7:3)/TB	0	28.7		146.7		6.5		165.0		19.3	
	7	36.1	46.6	149.6	153.2	28.2	26.6	166.5	168.5	17.8	14.8
	14	48.7	53.2	147.7	151.8	32.7	46.3	162.7	166.8	18.3	21.1
	28	44.5	52.5	135.5	144.4	37.9	53.6	152.5	165.5	23.3	17.6
PLA/PHB(6:4)	0	61.5		151.8		16.1		170.7		27.4	
	7	53.9	61.4	154.6	154.9	28.7	25.7	171.4	170.3	32.3	25.4
	14	44.4	55.1	153.6	155.0	38.4	32.5	168.6	170.7	34.0	29.3
	28	28.3	n.d.	138.2	144.2	39.0	35.7	156.9	168.3	35.7	35.7
PLA/PHB(6:4)/TB	0	28.5		146.4		9.6		165.3		23.4	
	7	33.4	47.7	149.9	153.5	23.2	28.2	167.3	169.3	25.8	24.9
	14	48.0	53.5	147.9	152.7	34.1	42.2	164.3	169.4	26.5	30.9
	28	34.4	n.d.	137.4	138.9	40.9	40.1	154.7	169.1	32.9	48.5

* ΔH_tot_ is the total enthalpy of each polymer, considering both the crystallization exotherm (when it occurs) and melting endotherm (ΔH_tot_ = Δ*H_m_* − Δ*H_c_*).

**Table 6:** TG and DTG parameters for annealed (A) and composted (C) films before and after 28 incubation days (values in parenthesis refer to temperature variation percentages with reference to time 0).

	[°C]	0 day	28 day	
			a	c
PLA	T_0_	322	213 (↓34%)	216 (↓33%)
	T_max,PLA_	363	319 (↓12%)	284 (↓22%)
PHB	T_0_	250	213 (↓15%)	226 (↓10%)
	T_max,PHB_	277	248 (↓11%)	249 (↓10%)
PLA/PHB(7:3)	T_0_	260	226 (↓13%)	219(↓16%)
	T_max,PHB_	278	278 (0%)	262 (↓6%)
	T_max,PLA_	352	316 (↓10%)	309 (↓12%)
PLA/PHB(7:3)/TB	T_0_	222	202 (↓9%)	222 (0%)
	T_max,PHB_	283	271 (↓4%)	252 (↓11%)
	T_max,PLA_	354	325 (↓8%)	294 (↓17%)
PLA/PHB(6:4)	T_0_	258	211 (↓18%)	220 (↓15%)
	T_max,PHB_	279	251 (↓10%)	245 (↓12%)
	T_max,PLA_	351	278 (↓21%)	277 (↓21%)
PLA/PHB(6:4)/TB	T_0_	208	198 (↓5%)	218 (↑5%)
	T_max,PHB_	278	248 (↓11%)	246 (↓12%)
	T_max,PLA_	358	277 (↓23%)	276 (↓23%)

## Data Availability

The data are available from the corresponding author upon request.
